# Correlation between age-friendly communities and post-stroke depression

**DOI:** 10.1016/j.pmedr.2025.103262

**Published:** 2025-09-30

**Authors:** Xiaofan Yuan, Yang Gao, Fan Yang, Hui Xu, Hong Chen

**Affiliations:** Department of General Practice, Sichuan Provincial People's Hospital, School of Medicine, University of Electronic Science and Technology of China, No.32, West second Section, 1st Ring Rd, Qingyang District, Chengdu, China

**Keywords:** Age-friendly community, Post-stroke depression, Elderly patients, Stroke, Correlation

## Abstract

**Objective:**

While adverse environmental factors are linked to depressive symptoms, the relationship between age-friendly communities (AFC) and post-stroke depression (PSD) remains unclear.

**Methods:**

This prospective cohort study enrolled elderly patients with stroke discharged from the neurology department between October 2022 and January 2024. At six months post-discharge, participants were assessed through structured telephone interviews using the AFC framework and the Hamilton Depression Scale (HAMD). Correlation and logistic regression analyses were used to evaluate the associations between AFC domains and PSD.

**Results:**

Among 335 participants (mean age = 73.12 ± 6.8 years; 53.1 % male), 144 were diagnosed with PSD, including mild (*n* = 52), moderate (*n* = 30), severe (*n* = 27), and extreme (*n* = 35) cases. AFC scores showed a negative correlation with PSD severity (*r* = −0.63, *P* < 0.01). Three AFC domains served as protective factors against PSD: housing (OR = 0.76), outdoor spaces and buildings (OR = 0.78), and civic participation and employment (OR = 0.70). An integrated model that combined these AFC domains with post-stroke functional status and social support reached an area under the curve (AUC) of 0.86.

**Conclusions:**

Enhancements in housing, outdoor areas, and civic engagement could reduce the PSD burden and promote the integration of AFC principles into stroke recovery strategies.

## Highlights

• We examined the link between post-stroke depression and age-friendly communities.
• This study discussed global aging issues and offered relevant recommendations.
• This study aims to prevent and lessen the severity of post-stroke depression.
• This study demonstrated depression severity links to age-friendly communities.

## 1 Introduction

The World Health Organization (WHO) emphasizes aging and urbanization as two major global trends shaping the world's population. Demographic data shows that the percentage of people aged 65 and older is rising faster than any other age group worldwide. By 2030, it is expected that one in five people globally will be elderly (over 65) (Beard et al., 2016a; Beard et al., 2016b). If the urgent needs of this growing demographic and the quickly changing disease patterns are not addressed promptly and effectively, the resulting economic and political consequences could be significant (Staudinger et al., 2016).

The concept of active aging was introduced at the Second World Assembly on Aging in Madrid in 2002, highlighting the importance of a comprehensive approach that combines social participation, security, and health as the three key pillars to ensure that older adults are fully integrated into society and adequately supported, especially during times of need (Report of the World Health Organization, 2002). Building on this, in 2007, the WHO and the American Association of Retired Persons proposed creating Age-Friendly Communities (AFC) and cities. These aim to promote health and well-being in both rural and urban areas, enabling more people to age with dignity and good health (World Health Organization, 2007a; World Health Organization, 2007b; Kim, 2022). However, the specific policies for constructing AFC tailored to the national and demographic contexts of various countries are still evolving.

Worldwide, 23 % of the disease burden is linked to health problems among people aged 60 and older, with cardiovascular and cerebrovascular diseases making up 30.3 % of this burden (Prince et al., 2015). Stroke continues to be the second leading cause of disability among the elderly, placing a substantial burden on families and societies, especially in low- and middle-income countries where disability rates are disproportionately elevated (Sousa et al., 2009; Sousa et al., 2010). Furthermore, stroke survivors face a higher risk of developing post-stroke depression (PSD), a condition worsened by decreased self-care and less social engagement after the disability. Evidence indicates that both depression and stroke occurrence increase with age, and the development of PSD significantly raises post-stroke mortality rates (Sousa et al., 2010; Hornsten et al., 2013; Rautio et al., 2018; Hackett et al., 2004). Therefore, it is essential to focus on reducing excessive disability, managing chronic illnesses, and decreasing premature death as primary strategies for promoting active aging and developing AFC. Furthermore, society must prioritize mental health among the elderly to ensure they can maintain dignity and independence in later years.

Although earlier studies have shown a link between poor housing conditions and depressive symptoms, suggesting these factors should be considered in community planning to prevent depression (Hackett et al., 2004; Hackett et al., 2008), there is still no research examining the connection between PSD and AFC. Therefore, this study aims to explore the relationship between AFC and PSD to address this knowledge gap. The findings could provide theoretical insights for developing AFC and offer improved mental health support for stroke survivors.

## 2 Methods

### 2.1 Case selection

This study involved elderly patients newly diagnosed with stroke who were admitted to the neurology department at Sichuan Provincial People's Hospital between October 2022 and January 2024. The study complied with the institution's guidelines for protecting human subjects regarding safety and privacy (Approval No. 2022–472). Participants were selected based on their discharge diagnosis of either ischemic or hemorrhagic stroke. Six months after discharge, patients were evaluated through telephone follow-ups using the AFC index and the Hamilton Depression Scale (HAMD). Two well-trained researchers independently assigned scores for AFC and HAMD, and any disagreements were resolved by a third, senior reviewer.

The inclusion and exclusion criteria for participation in the study were as follows: Inclusion Criteria were (I) a confirmed diagnosis of ischemic or hemorrhagic stroke, verified according to the relevant clinical guidelines (Group of Cerebrovascular diseases, 2018; *Chinese Journal of Neurology*, 2019). (II) Age 60 years or older. (III) Signed informed consent to participate in the study. Exclusion Criteria: (I) A history of depression, either untreated or unresolved after psychiatric evaluation, before the current hospitalization. (II) Severe cognitive impairment or speech difficulties that hinder the completion of the necessary assessments. (III) Severe neurological deficits or unstable vital signs at stroke onset. (IV) A personal or family history of psychiatric disorders.

### 2.2 Diagnosis of PSD

The diagnosis of PSD was made based on established criteria in China (Wang Shaoshi and Xinyu, 2016). Depression severity was measured using the HAMD, with the following classification: (I) A score less than 7 indicates normal mood. (II) A score between 7 and 17 signifies mild depression. (III) A score between 17 and 24 indicates moderate depression. (IV) A score between 24 and 30 means severe depression. (V) A score greater than 30 is classified as extreme depression.

### 2.3 Assessment of AFC

The AFC level was assessed using a scale based on WHO guidelines (Kim, 2022), covering eight main areas: housing, transportation, outdoor spaces and buildings, community support and health services, communication and information, social participation, respect and social inclusion, and civic participation and employment. Each area was scored on a five-point scale: 1 for strongly disagree, 2 for disagree, 3 for neutral, 4 for agree, and 5 for strongly agree. Higher AFC scores reflect a more age-friendly environment for the elderly patients with stroke.

### 2.4 Demographic and clinical data

The following demographic and clinical data were collected: Demographic information included age, sex, household income per capita, antidepressant use, type of stroke (ischemic or hemorrhagic), and stroke location (such as frontal stroke). Medical History encompassed Body Mass Index (BMI), lifestyle habits (smoking, alcohol consumption), and comorbidities (diabetes, hypertension, coronary artery disease, atrial fibrillation). Clinical Parameters involved laboratory values such as fasting blood glucose (FBG), uric acid, triglycerides, total cholesterol, low-density lipoprotein (LDL), high-density lipoprotein (HDL), along with clinical assessments using the National Institutes of Health Stroke Scale (NIHSS), modified Rankin Scale (mRS), Perceived Social Support Scale (PSSS), AFC, and HAMD scores.

### 2.5 Statistical analysis

Continuous data with normal distribution is expressed as mean ± standard deviation (χ ± s) and compared using a *t*-test or Analysis of Variance (ANOVA). Data with non-normal distribution are expressed as medians and compared using the Wilcoxon rank-sum test. Categorical data are expressed as frequencies and percentages, and compared using the χ2 test. The Spearman correlation was used to evaluate the relationship between AFC and PSD. Variables with *p* < 0.01 in univariate analysis were included in the multivariable logistic regression model. A further multivariable logistic regression was conducted to assess the association between multi-dimensional AFC and PSD. *P* < 0.05 was considered statistically significant. SPSS version 26.0 (SPSS, Chicago, IL) and GraphPad Prism version 9.0 (GraphPad Software, San Diego, California, USA) were used for statistical analysis.

## 3 Results

A total of 404 patients were initially enrolled in this study. After excluding 69 cases due to incomplete data or inability to complete the assessment scales, 335 patients were included in the final analysis. Of these, 178 were male and 157 were female, with a mean age of 73.12 years (ranging from 60 to 92). Among the 335 participants, 144 were diagnosed with PSD based on the scores of HAMD. The depression severity was categorized as mild (52 patients), moderate (30 patients), severe (27 patients), and extreme (35 patients) respectively. Additionally, we compared baseline characteristics between the 335 completers and the 69 dropouts. No significant differences were observed in age, sex, stroke type, stroke location, or baseline NIHSS scores between the two groups (*p* > 0.05).

### 3.1 Demographic and clinical data

Patients were classified into non-PSD and PSD groups based on their HAMD scores. Statistical analysis showed no significant differences between the non-PSD and PSD groups regarding age, sex, BMI, household income, stroke type, stroke location, lifestyle habits, comorbidities, FBG, uric acid, triglycerides, total cholesterol, HDL, LDL, and NIHSS scores. The use of antidepressants, as well as scores of mRS and PSSS, showed significant differences between non-PSD and PSD patients, as shown in Table 1.

**Table 1:** The demographic and clinical data of patients with and without post-stroke depression discharged from the neurology department of China between October 2022 and January 2024.

Variables	Non-PSD n(%)/mean(SD)	PSD n(%)/mean(SD)	P value
Age	73.98 ± 6.38	72.83 ± 7.32	0.52
Male	98 (51.30)	80 (55.56)	0.71
BMI	22.73 ± 3.63	23.81 ± 3.08	0.82
Smoking	70 (36.65)	62 (43.06)	0.62
Drinking	92 (48.17)	81 (56.25)	0.09
Hypertension	142 (74.35)	112 (77.78)	0.12
Diabetes	96 (50.26)	85 (59.03)	0.26
Coronary heart disease	74 (38.74)	67 (46.53)	0.19
Atrial fibrillation	17 (8.90)	12 (8.33)	0.38
Ischemic stroke	152 (79.58)	120 (83.33)	0.85
FBG	9.68 ± 3.29	10.61 ± 4.01	0.74
Uric acid	402.63 ± 18.27	398.94 ± 19.02	0.62
Total cholesterol	3.79 ± 1.35	3.53 ± 1.47	0.09
Triglycerides	1.85 ± 0.64	1.90 ± 0.58	0.28
HDL	1.09 ± 0.34	1.16 ± 0.46	0.83
LDL	2.47 ± 0.81	2.28 ± 0.79	0.45
mRS	1.57 ± 0.61	2.12 ± 0.87	<0.01
PSSS	42.49 ± 10.12	34.11 ± 9.81	<0.01
Frontal stroke	52 (27.23)	42 (29.17)	0.29
NIHSS	6.83 ± 2.46	7.02 ± 1.98	0.39
Antidepressant use	6 (3.14)	41 (28.47)	<0.01
household income	7892 ± 1103	7918 ± 912	0.47

PSD, post-stroke depression; BMI, body mass index; FBG, fasting blood glucose; HDL, high-density lipoprotein; LDL, low-density lipoprotein; SD, Standard Deviation; mRS, modified Rankin Scale; PSSS, Perceived Social Support Scale; NIHSS, National Institutes of Health Stroke Scale. Scale interpretations: mRS: Range 0–6, higher score = greater disability. PSSS: Range 12–84, higher score = greater perceived social support. NIHSS: Range 0–42, higher score = more severe nerve injury. *P* values were calculated using an independent samples *t*-test for continuous variables, expressed as mean ± SD; a Mann-Whitney *U* test for non-normally distributed continuous variables; and a χ^2^ test for categorical variables, expressed as n (%).

### 3.2 AFC assessment

**Fig. 1:**
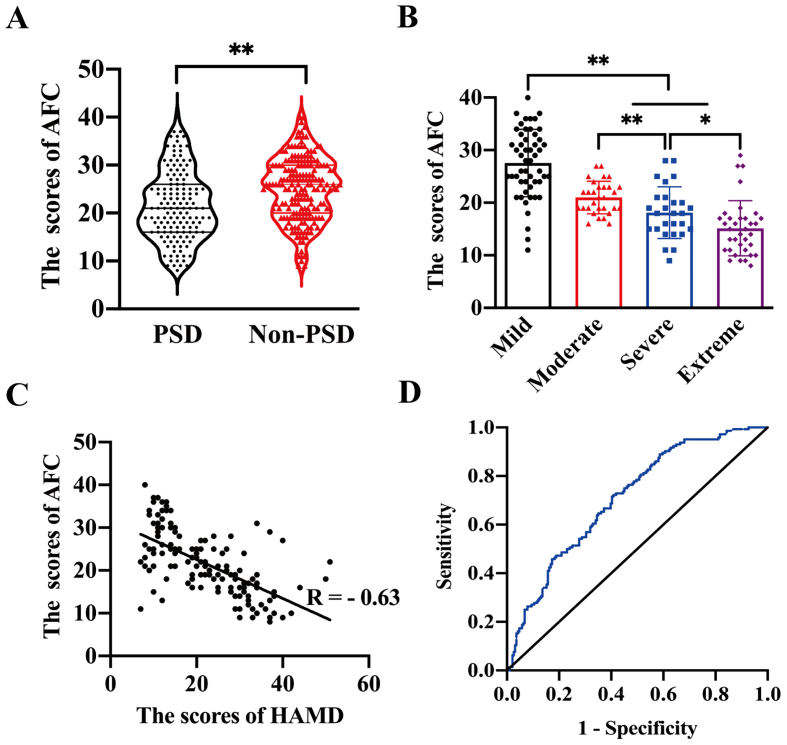
Evaluation of age-friendly community scores in patients with and without post-stroke depression (A), and patients with post-stroke depression were divided by depression severity (B). The relationship between scores of age-friendly community and Hamilton depression scale in patients with post-stroke depression is shown in (C). The Receiver Operating Characteristic curve analysis assesses age-friendly community and clinical factors for post-stroke depression prediction (D). Participants were discharged from the neurology department of China between October 2022 and January 2024. 1 A: The AFC scores in patients diagnosed with PSD (21.50 ± 7.25) and Non-PSD (25.08 ± 6.59). **P < 0.01. *P* values were calculated using an independent samples *t*-test. 1B: Comparison of AFC scores among patients with different levels of PSD. AFC scores decreased progressively with worsening depression categories, and the differences were statistically significant. *P < 0.05, **P < 0.01. P values were calculated using ANOVA. 1C: There is a negative correlation between AFC scores and PSD severity, **P < 0.01. P values were obtained using Spearman's correlation analysis. 1D: The multivariable model combining multidimensional AFC domains (housing, outdoor spaces and buildings, and civic participation and employment) with clinically relevant factors (mRS and PSSS) demonstrated improved predictive performance for PSD, with an AUC of 0.86, compared to the AFC-only model (AUC = 0.71) and the clinical factors-only model (AUC = 0.65). The combined model achieved a sensitivity of 81.4 % and a specificity of 86.3 %. AFC, age-friendly community; PSD, post-stroke depression; HAMD, Hamilton Depression Scale; mRS, modified Rankin Scale; PSSS, Perceived Social Support Scale. Scale interpretations: mRS ranges from 0 to 6, with higher scores indicating greater disability. PSSS ranges from 12 to 84, with higher scores representing greater perceived social support. Total AFC domain scores range from 8 to 40, scored 1–5 per item; higher scores reflect a more age-friendly community. HAMD scores range from 0 to 52, with higher scores indicating more severe depression (Normal <7; Mild = 7–17; Moderate = 17–24; Severe = 24–30; Extreme>30).

Patients with a HAMD score below seven were classified as not having PSD, and their AFC scores were significantly higher compared to those diagnosed with PSD (25.08 ± 6.59 vs. 21.50 ± 7.25, *P* < 0.01), as shown in Fig. 1A. Further analysis of AFC scores among PSD patients with different levels of depression severity revealed a clear inverse trend: as depression severity increased, AFC scores decreased (mild > moderate > severe > extreme; *P* < 0.05), as depicted in Fig. 1B. And the internal consistency (Cronbach's α = 0.89) and test-retest reliability (Intraclass Correlation Coefficient = 0.85) demonstrated good validity of the AFC scale in this study.

### 3.3 Correlation analysis between AFC and PSD

Spearman's correlation analysis showed a significant negative correlation between AFC scores and the occurrence of PSD in elderly patients (*r* = −0.63, *P* < 0.05), as illustrated in Fig. 1C. This indicates that elderly individuals living in less age-friendly environments are at a higher risk of developing PSD. Lower AFC scores in PSD patients are associated with greater severity of depression symptoms.

### 3.4 Logistic regression analysis of multi-dimensional AFC factors affecting PSD

To further examine the impact of various AFC dimensions on PSD, we performed logistic regression analysis. In the univariate logistic regression, besides the six AFC dimensions (housing, social participation, outdoor spaces and buildings, communication and information, civic participation and employment, community support and health services), the mRS and PSSS were also identified as protective factors for PSD. The multivariable logistic regression analysis in Table 2, which incorporated the statistically significant indicators from the univariate analysis, identified five key variables as independent protective factors against PSD in the elderly. These were mRS (OR = 0.77), PSSS (OR = 0.80), housing (OR = 0.76), outdoor spaces and buildings (OR = 0.78), and civic participation and employment (OR = 0.70).

**Table 2:** Logistic regression analysis of age-friendly communities in patients with post-stroke depression discharged from the neurology department of China between October 2022 and January 2024.

Variables	Univariate logistic regression		Multivariable logistic regression	
Factors	OR (95 % CI)	*P* value	OR (95 % CI)	P value
mRS	0.88 (0.79, 0.94)	<0.01	0.77 (0.67, 0.82)	<0.01
PSSS	0.84 (0.78, 0.90)	<0.01	0.80 (0.71, 0.86)	<0.01
Housing	0.68 (0.56, 0.81)	<0.01	0.76 (0.59, 0.97)	0.03
Transportation	0.85 (0.70, 1.01)	0.07		
Social participation	0.77 (0.63, 0.94)	<0.01		
Outdoor spaces and buildings	0.71 (0.60, 0.83)	<0.01	0.78 (0.64, 0.95)	0.01
Social inclusion and respect	0.87 (0.74, 1.02)	<0.01		
Communication and information	0.82 (0.68, 0.98)	0.03		
Civic participation and employment	0.67 (0.57, 0.78)	<0.01	0.70 (0.58, 0.84)	<0.01
Community support and health services	0.85 (0.72, 1.10)	0.05		

AFC, age-friendly community; PSD, post-stroke depression; mRS, modified Rankin Scale; PSSS, Perceived Social Support Scale; OR, odds ratios; CI, confidence interval. Scale interpretations: mRS ranges from 0 to 6, with higher scores indicating greater disability. PSSS ranges from 12 to 84, with higher scores indicating greater perceived social support. The total AFC score covers eight domains (housing, transportation, social participation, outdoor spaces and buildings, social inclusion and respect, communication and information, civic participation and employment, community support and health services): scored 1–5 per item, with a total range of 8–40; higher scores reflect a more age-friendly community. *P*-value and OR were calculated using univariate and multivariable logistic regression analyses.

### 3.5 Predictive value of multi-dimensional AFC factors for PSD

Our study on PSD predictors found that multi-dimensional AFC factors, covering outdoor spaces and buildings, civic participation and employment, and housing were significant predictors, with an area under the curve (AUC) of 0.71. Moreover, clinically relevant factors such as mRS and PSSS also showed moderate predictive power for PSD, with an AUC of 0.65. To further improve predictive accuracy, we integrated AFC variables with clinically relevant factors. This combined predictive model showed significantly better performance, with an AUC of 0.86, and sensitivity and specificity of 89.5 % and 88.4 %,respectively (Fig. 1D). These findings highlight the potential of interventions targeting both specific aspects of AFC and key clinical factors, such as functional status and social support, to reduce the incidence of PSD.

## 4 Discussion

According to the 2019 Global Burden of Disease study, stroke is the second leading cause of death and the third leading cause of disability-adjusted life years worldwide. The total number of stroke-related disabilities continues to increase each year globally (GBD 2019 Stroke Collaborators, 2021). Beyond physical and cognitive impairments, about one-third of stroke survivors face emotional disturbances, including symptoms such as emotional instability, anhedonia, anxiety, and depression (Ayerbe et al., 2013). The pathophysiology of PSD is thought to involve dysregulation of the hypothalamic-pituitary-adrenal axis, abnormal neurotrophic responses, increased inflammatory cytokine levels, decreased excitotoxicity mediated by glutamate, and altered monoamine oxidase activity (Guo et al., 2022). Notably, elderly patients with PSD show significant impairments in functional rehabilitation, lower medication adherence, reduced social participation, and a higher risk of recurrent stroke, rehospitalization, and overall mortality compared to younger stroke survivors (Masuccio et al., 2024). Therefore, the diagnostic criteria and treatment methods for PSD in older adults need further refinement to better address the specific challenges faced by this population.

In this study, about 40.56 % of elderly patients with stroke were diagnosed with PSD. Among these, mild depression was the most common, affecting roughly 36.11 % of patients. The rate of PSD in our group was significantly higher than that reported in earlier studies (Ayerbe et al., 2013; Guo et al., 2022; Shi et al., 2017; Karakus et al., 2017; Yang et al., 2013). We hypothesize that this higher incidence may be due to factors such as decreased self-care ability and mobility in elderly stroke survivors, which limit social interactions and raise the risk of depression. Furthermore, differences in the assessment scales for PSD and the severity of stroke across studies may also contribute to the observed discrepancies in prevalence.

Our findings also revealed a significant negative correlation between the AFC index and the occurrence of PSD. Specifically, lower AFC scores were linked to increased depression severity in elderly patients. Stroke survivors, especially older adults, often experience greater dependence on their families and communities. The environmental friendliness of their living conditions is essential for their health and well-being. Living in communities with more age-friendly infrastructure and a better match between individuals and their environment has been shown to enhance quality of life for the elderly (Choi, 2020; Kim et al., 2022). A high level of community engagement indicates that elderly people feel more satisfied with their lives and participate more in activities, which is a crucial part of AFC (Zheng et al., 2020; Liao et al., 2022; Zhang et al., 2020; Tsuji et al., 2022). Therefore, governments and society must actively raise awareness of the importance of age-friendly communities and support the creation of environments that can help prevent PSD and lower the severity of depression levels.

Multivariable logistic regression further showed that key AFC dimensions, such as housing, outdoor spaces and buildings, civic participation, and employment, are independent risk factors for PSD. Combining these AFC dimensions with clinically relevant factors, such as mRS and PSSS, resulted in an AUC of 0.86, indicating that future research should aim to develop more comprehensive predictive tools that incorporate biomedical, psychological, and environmental factors. Additionally, earlier studies have demonstrated a negative correlation between depression and social participation, showing that increased social engagement can lead to reduced depressive symptoms (Tse et al., 2017; Chen et al., 2018; Tse et al., 2019). .Furthermore, lack of social activity and depressive symptoms remain common even years after stroke recovery, highlighting the importance of ongoing social engagement to prevent long-term depression (Mutai et al., 2013). Based on these findings, we suggest that in addition to traditional pharmacological and psychological treatments, a comprehensive assessment of AFC related to social participation, housing, and transportation should be included in care plans to support the mental health and independence of elderly stroke patient survivors.

The increased dependence of elderly patients with PSD on their surroundings underscores the importance of creating age-friendly environments. Good infrastructure, such as accessible buildings, reliable transportation, and numerous social opportunities, can enhance social participation and relationships, contributing to successful aging in place. We support ongoing efforts by governments to develop high-quality, accessible community settings that enable people with PSD to become more integrated into their communities and gain from AFC principles.

## 5 Limitations

This study has several limitations. First, follow-up assessments were conducted via telephone rather than face-to-face interviews, which may have introduced data bias, especially among the elderly population. Second, the follow-up period was limited to six months post-discharge, and long-term changes in AFC and PSD require further research. Additionally, future studies should aim to evaluate the long-term effects of AFC on the mental health of elderly patients with stroke and explore the potential for sustained interventions to prevent PSD over time.

## 6 Conclusion

This study shows that the AFC index is negatively linked to the occurrence of PSD in elderly patients. Those living in less age-friendly environments are more likely to develop severe depressive symptoms. Improving community factors such as housing, outdoor spaces and buildings, civic participation and employment may help prevent PSD and lessen depression severity in older adult populations.

## Informed consents and ethics approval

All of patients participated in this study signed informed consents. And this study was approved by the Ethics Committee of Sichuan Provincial People's Hospital (Approval No. 2022–472).

## CRediT authorship contribution statement

**Xiaofan Yuan:** Project administration, Funding acquisition, Formal analysis, Data curation. **Yang Gao:** Writing – original draft, Visualization, Resources, Data curation. **Fan Yang:** Writing – original draft, Validation, Supervision, Resources. **Hui Xu:** Writing – original draft, Validation, Methodology, Conceptualization. **Hong Chen:** Writing – review & editing, Visualization, Supervision, Formal analysis.

## Compliance with ethical standards

We have complied with APA ethical principles in our treatment of individuals participating in the study. All of patients participated in this study signed informed consents. And this study was approved by the Ethics Committee of Sichuan Provincial People's Hospital, School of Medicine, University of Electronic Science and Technology of China.

## Funding

This study is supported by Sichuan Science and Technology Program (No. 2024NSFSC0622).

## Declaration of competing interest

The authors declare that they have no known competing financial interests or personal relationships that could have appeared to influence the work reported in this paper.

## Data Availability

Data will be made available on request.
